# Supplementary material for: A synergy-based hand control is encoded in human motor cortical areas
Source: eLife. 2016 Feb 15;5:e13420. doi: 10.7554/eLife.13420 (PMC4786436; doi:10.7554/eLife.13420)
Supplement: Supplementary file 1. — Values of rank accuracy, measured with the leave-one-stimulus-out procedure, for the nine subjects, with the p-value obtained from the permutation test (10000 iterations). The comparison between the performance values indicate that the kinematic synergy model was significantly better than both the individual digit and muscle synergy models (Wilcoxon signed-rank test, p=0.0078), and the individual digit model was significantly more informative than the muscle synergy model (p=0.0156). (B) Single subject encoding accuracy values. The accuracy of predicting brain activity from the behavioral models (kinematic synergy, individual digit and muscle synergy models), obtained with the cross-validation procedure, is reported here for each subject, along with the chance levels derived from the permutation tests, the threshold at p=0.05 and the actual p-value obtained from the tests against the null distributions of accuracies. The accuracy values reported in red are not significant. The comparisons between individual accuracy values, performed using Wilcoxon signed-rank tests, show that the kinematic synergy model outperformed both the individual digit (p=0.0234) and the muscle synergy (p=0.0391) models, whereas no significant difference was found between the individual digit and muscle synergy models (p=0.9453). (C) Size and coordinates of the clusters of greatest overlap between subjects. This table reports the regions that were consistently recruited across subjects (p>0.33, 4 out of 9 subjects). The region names are reported alongside with their size and with the coordinates of the peak voxel in RAI orientation according to the MNI 152 atlas. (D) RSA results: single-subject and group correlations between RSs. The table contains the results from Representational Similarity Analysis (RSA). The single-subject correlation values are reported, along with the group-level correlation (i.e. obtained from the averaged RSs across subjects) and with the p-values resulting from the M [file elife-13420-supp1.docx]

**Supplementary File 1A: Single subject rank accuracy values**

|  | *kinematic synergy model* | |  | *individual digit model* | |  | *muscle synergy model* | |
| --- | --- | --- | --- | --- | --- | --- | --- | --- |
| Subject | Accuracy | *p*-value |  | Accuracy | *p*-value |  | Accuracy | *p*-value |
| 01 | 92.24 | <0.0001 |  | 86.27 | <0.0001 |  | 58.03 | 0.0012 |
| 02 | 92.15 | <0.0001 |  | 85.88 | <0.0001 |  | 74.69 | <0.0001 |
| 03 | 94.43 | <0.0001 |  | 90.7 | <0.0001 |  | 74.61 | <0.0001 |
| 04 | 93.9 | <0.0001 |  | 90.09 | <0.0001 |  | n.a. | n.a. |
| 05 | 83.16 | <0.0001 |  | 75.11 | <0.0001 |  | 76.75 | <0.0001 |
| 06 | 88.95 | <0.0001 |  | 82.05 | <0.0001 |  | 73.73 | <0.0001 |
| 07 | 93.89 | <0.0001 |  | 90.58 | <0.0001 |  | 62.06 | <0.0001 |
| 08 | 92.84 | <0.0001 |  | 88 | <0.0001 |  | 76.14 | <0.0001 |
| 09 | 88.74 | <0.0001 |  | 84.05 | <0.0001 |  | 80.04 | <0.0001 |
|  |  |  |  |  |  |  |  |  |
| AVG | 91.14 |  |  | 85.86 |  |  | 72.01 |  |
| SD | 3.63 |  |  | 5.02 |  |  | 7.70 |  |

**Supplementary File 1B: Single subject encoding accuracy values**

|  | *kinematic synergy model* | | | |  | *individual digit model* | | | |  | *muscle synergy model* | | | |
| --- | --- | --- | --- | --- | --- | --- | --- | --- | --- | --- | --- | --- | --- | --- |
| subject | accuracy | chance | threshold | *p*-value |  | accuracy | chance | threshold | *p*-value |  | accuracy | chance | threshold | *p*-value |
| 01 | 68.16 | 49.86 | 62.90 | 0.008 |  | 59.74 | 49.78 | 61.58 | 0.087 |  | 58.42 | 49.85 | 62.37 | 0.136 |
| 02 | 81.58 | 49.73 | 61.05 | <0.001 |  | 71.58 | 50.19 | 61.84 | <0.001 |  | 66.84 | 50.01 | 61.58 | 0.011 |
| 03 | 72.63 | 49.93 | 61.05 | <0.001 |  | 76.32 | 50.37 | 62.63 | <0.001 |  | 67.11 | 49.83 | 61.05 | 0.008 |
| 04 | 66.32 | 50.31 | 62.63 | 0.008 |  | 62.37 | 50.07 | 62.37 | 0.048 |  | n.a. | n.a. | n.a. | n.a. |
| 05 | 79.21 | 50.01 | 61.32 | <0.001 |  | 58.68 | 50.06 | 61.32 | 0.095 |  | 61.84 | 49.74 | 61.05 | 0.038 |
| 06 | 70.26 | 50.03 | 61.05 | 0.001 |  | 56.58 | 50.14 | 60.79 | 0.152 |  | 65.26 | 49.96 | 60.00 | 0.008 |
| 07 | 72.11 | 49.96 | 61.05 | <0.001 |  | 66.84 | 50.31 | 61.57 | 0.008 |  | 67.63 | 50.03 | 62.11 | 0.01 |
| 08 | 66.32 | 50.03 | 60.79 | 0.001 |  | 56.84 | 49.88 | 60.26 | 0.118 |  | 51.84 | 49.90 | 60.53 | 0.384 |
| 09 | 67.63 | 50.00 | 63.16 | 0.013 |  | 66.05 | 49.88 | 62.9 | 0.021 |  | 72.90 | 50.00 | 62.90 | 0.001 |
|  |  |  |  |  |  |  |  |  |  |  |  |  |  |  |
| AVG | 71.58 | 49.99 | 61.67 |  |  | 63.89 | 50.08 | 61.69 |  |  | 63.98 | 49.92 | 61.45 |  |
| SD | 5.52 | 0.16 | 0.94 |  |  | 6.86 | 0.20 | 0.85 |  |  | 6.49 | 0.10 | 0.97 |  |

**Supplementary File 1C: Size and coordinates of the clusters of greatest overlap between subjects**

|  |  | *x* | *y* | *z* |  | *Voxels* |  | Region |
| --- | --- | --- | --- | --- | --- | --- | --- | --- |
| 1 |  | 36 | 30 | 50 |  | 1701 |  | Left Precentral, Postcentral, Superior, & Inferior Parietal cortex |
| 2 |  | -38 | 32 | 42 |  | 68 |  | Right Postcentral Cortex |
| 3 |  | 4 | 8 | 52 |  | 59 |  | Left Supplementary Motor Area |
| 4 |  | 54 | 26 | 36 |  | 18 |  | Left Inferior Parietal Lobule |
| 5 |  | -48 | 26 | 42 |  | 16 |  | Right Postcentral Cortex |
| 6 |  | 56 | -6 | 32 |  | 12 |  | Left Brodmann Area 6 |
| 7 |  | -62 | -6 | 34 |  | 6 |  | Right Brodmann Area 6 |

**Supplementary File 1D: RSA results: single-subject and group correlations between RSs**

|  | kinematic-ID | | kinematic-EMG | | ID-EMG | |
| --- | --- | --- | --- | --- | --- | --- |
| subject | correlation | *p*-value | correlation | *p*-value | correlation | *p*-value |
| 01 | 0.8974 | 0.0001 | 0.5972 | 0.0001 | 0.5964 | 0.0001 |
| 02 | 0.8182 | 0.0001 | 0.4956 | 0.0001 | 0.5113 | 0.0001 |
| 03 | 0.8959 | 0.0001 | 0.6281 | 0.0001 | 0.6051 | 0.0001 |
| 04 | 0.9163 | 0.0001 | n.a. | n.a. | n.a. | n.a. |
| 05 | 0.8072 | 0.0001 | 0.3331 | 0.0001 | 0.2054 | 0.0014 |
| 06 | 0.9378 | 0.0001 | 0.4749 | 0.0001 | 0.4523 | 0.0001 |
| 07 | 0.7377 | 0.0001 | 0.3755 | 0.0001 | 0.2286 | 0.0007 |
| 08 | 0.7917 | 0.0001 | 0.3237 | 0.0001 | 0.1182 | 0.557 |
| 09 | 0.8954 | 0.0001 | 0.3094 | 0.0001 | 0.1914 | 0.0042 |
|  |  |  |  |  |  |  |
| group | 0.9236 | 0.0001 | 0.7655 | 0.0001 | 0.6912 | 0.0001 |

**Supplementary File 1E: RSA results: single-subject and group correlations between behavioral and fMRI RSs**

|  | fMRI-kinematic | | fMRI-ID | | fMRI-EMG | |
| --- | --- | --- | --- | --- | --- | --- |
| subject | correlation | *p*-value | correlation | *p*-value | correlation | *p*-value |
| 01 | 0.3345 | 0.0001 | 0.3321 | 0.0001 | 0.0351 | 0.3097 |
| 02 | 0.2962 | 0.0001 | 0.2259 | 0.0004 | 0.119 | 0.0469 |
| 03 | 0.1448 | 0.0022 | 0.1833 | 0.0005 | 0.2609 | 0.0001 |
| 04 | 0.1906 | 0.0046 | 0.2097 | 0.0025 | n.a. | n.a. |
| 05 | 0.4277 | 0.0001 | 0.2965 | 0.0001 | -0.0157 | 0.5846 |
| 06 | 0.4059 | 0.0001 | 0.4767 | 0.0001 | 0.2532 | 0.0001 |
| 07 | 0.1929 | 0.004 | 0.2719 | 0.0001 | -0.025 | 0.6325 |
| 08 | 0.3613 | 0.0001 | 0.1998 | 0.0034 | 0.4027 | 0.0001 |
| 09 | 0.3287 | 0.0001 | 0.2818 | 0.0003 | 0.2919 | 0.0001 |
|  |  |  |  |  |  |  |
| group | 0.6036 | 0.0001 | 0.5413 | 0.0001 | 0.5839 | 0.0001 |

**Supplementary File 1F: Goodness of fit between original and decoded hand postures**

| *object* | *R^2^* | *STD* |  | *object* | *R^2^* | *STD* |
| --- | --- | --- | --- | --- | --- | --- |
| Pen | 0.9025 | 0.0391 |  | Light bulb | 0.7636 | 0.1806 |
| Tennis Racket | 0.8773 | 0.0424 |  | Toothpick | 0.7611 | 0.1534 |
| Ice cube | 0.8666 | 0.0642 |  | Espresso cup | 0.7430 | 0.1020 |
| Telephone handset | 0.8630 | 0.1122 |  | Wrench | 0.7048 | 0.2539 |
| Hammer | 0.8611 | 0.0574 |  | Bucket | 0.6746 | 0.2726 |
| Fishing rod | 0.8559 | 0.1333 |  | Jar lid | 0.6400 | 0.3225 |
| Cherry | 0.8277 | 0.0691 |  | Dinner plate | 0.5956 | 0.2040 |
| Chalk | 0.8013 | 0.1237 |  | Frisbee | 0.5838 | 0.2725 |
| Hair dryer | 0.7981 | 0.1742 |  | PC mouse | 0.5470 | 0.1842 |
| Rope | 0.7849 | 0.1290 |  | Calculator | 0.5099 | 0.4614 |

**Supplementary File 1G: Rank accuracy values between original and decoded hand postures**

| subject | Accuracy (%) | SEM | *p*-value |
| --- | --- | --- | --- |
| 01 | 59.21 | 6.87 | 0.0585 |
| 02 | 70.26 | 6.47 | 0.0006 |
| 03 | 71.05 | 4.56 | 0.0005 |
| 04 | 67.37 | 6.37 | 0.0027 |
| 05 | 67.63 | 6.04 | 0.0021 |
| 06 | 72.11 | 4.15 | 0.0004 |
| 07 | 52.37 | 7.04 | 0.2876 |
| 08 | 62.63 | 6.83 | 0.0139 |
| 09 | 54.74 | 6.97 | 0.1816 |

**Supplementary File 1H: Encoding accuracy values for the picture-related brain activity**

|  | *kinematic synergy model* | | | |
| --- | --- | --- | --- | --- |
| subject | accuracy | chance | threshold | *p*-value |
| 01 | 52.895 | 49.888 | 58.947 | 0.268 |
| 02 | 53.421 | 49.776 | 56.842 | 0.164 |
| 03 | 56.316 | 49.977 | 58.947 | 0.112 |
| 04 | 53.421 | 50.242 | 60.526 | 0.29 |
| 05 | 55.263 | 50.159 | 59.737 | 0.201 |
| 06 | 61.316 | 50.008 | 62.105 | 0.062 |
| 07 | 58.158 | 49.754 | 59.211 | 0.063 |
| 08 | 41.316 | 49.941 | 62.015 | 0.883 |
| 09 | 43.684 | 49.794 | 58.947 | 0.884 |
|  |  |  |  |  |
| AVG | 52.87 | 49.95 | 59.70 |  |
| SD | 6.47 | 0.17 | 1.66 |  |

**Supplementary File 1I: Encoding accuracy values for kinematic synergies in visual areas**

|  | *kinematic synergy model* | | | |
| --- | --- | --- | --- | --- |
| subject | accuracy | chance | threshold | *p*-value |
| 01 | 52.105 | 49.582 | 58.684 | 0.29 |
| 02 | 42.368 | 51.887 | 61.053 | 0.99 |
| 03 | 59.737 | 49.932 | 61.316 | 0.084 |
| 04 | 50.789 | 49.816 | 58.421 | 0.44 |
| 05 | 68.158 | 50.455 | 61.053 | 0.01 |
| 06 | 59.474 | 49.997 | 60 | 0.073 |
| 07 | 58.947 | 49.766 | 59.737 | 0.063 |
| 08 | 56.579 | 50.979 | 61.053 | 0.2 |
| 09 | 65.789 | 50.058 | 60.263 | 0.02 |
|  |  |  |  |  |
| AVG | 57.11 | 50.27 | 60.18 |  |
| SD | 7.42 | 0.69 | 1 |  |

**Supplementary File 1J: List of objects**

| 1. | Bucket |
| --- | --- |
| 2. | Calculator |
| 3. | Chalk |
| 4. | Cherry |
| 5. | Dinner plate |
| 6. | Espresso cup |
| 7. | Fishing rod |
| 8. | Frisbee |
| 9. | Hammer |
| 10. | Hair dryer |
| 11. | Ice cube |
| 12. | Jar lid |
| 13. | Light bulb |
| 14. | PC mouse |
| 15. | Pen |
| 16. | Rope |
| 17. | Telephone handset |
| 18. | Tennis racket |
| 19. | Toothpick |
| 20. | Wrench |

**Supplementary File 1K: List of marked joints and bones**

**Joints:**

Thumb carpo-metacarpal (CMC)

Thumb metacarpophalangeal (MCP)

Thumb interphalangeal (IP)

Index metacarpophalangeal (MCP)

Index proximal interphalangeal (PIP)

Middle finger metacarpophalangeal (MCP)

Middle finger proximal interphalangeal (PIP)

Ring finger proximal interphalangeal (PIP)

Little finger proximal interphalangeal (PIP)

**Bones:**

Thumb metacarpal bone (MC)

Thumb proximal phalanx (PP)

Thumb distal phalanx (DP)

Index metacarpal bone (MC)

Index proximal phalanx (PP)

Index distal phalanx (DP)

Middle finger metacarpal bone (MC)

Middle finger proximal phalanx (PP)

Middle finger distal phalanx (DP)

Ring finger metacarpal bone (MC)

Ring finger proximal phalanx (PP)

Ring finger distal phalanx (DP)

Little finger metacarpal bone (MC)

Little finger proximal phalanx (PP)

Little finger distal phalanx (DP)

**Supplementary File 1L: EMG features**

1. *Median frequency:*

It is the frequency value for which the sum of all power densities for lower frequencies equals the sum of all power densities for higher frequencies.

2 *Mean frequency:*

Power-density-weighted mean frequency.

3 *Spectral deformation:*

Estimated below 1000 Hz, it is the ratio between the square root of the squared-power-density-weighted mean frequency and the mean frequency (Sinderby et al., 1995).

4 *Signal to noise ratio (SNR):*

Estimated below 1000 Hz, it is the ratio between the sum of all power densities and the noise, estimated as the average of all power densities in the upper 20% frequency range (Sinderby et al., 1995).

5 *Maximum to minimum drop in power density ratio (DPR):*

Estimating mean power density as the average of 13 consecutive points in the EMG power spectrum, the DPR is the ratio between the highest and the lowest mean power densities (Sinderby et al., 1995).

6 *Signal-to-motion artifact ratio (SMR):*

Two assumptions are formulated to estimate such a feature: (1) frequency of motion artifacts are below 20 Hz and (2) the shapes of the non-contaminated EMG power spectrum is fairly linear below 20 Hz. SMR is therefore estimated as the ratio of the sum of all power densities for all frequencies below 600 Hz and the sum of all power densities below 20 Hz that exceed a straight line between the axis origin and the highest mean power density above 35 Hz. The mean power density is estimated as the average of 13 consecutive points in the EMG power spectrum (Sinderby et al., 1995).

7 *First Autoregressive (AR) coefficient:*

First coefficient of the fourth order autoregressive model. EMG signals are described as linear combinations of previous samples plus a white noise error term, using the Levinson-Durbin algorithm.

8 *Second Autoregressive (AR) coefficient:*

Second coefficient of the fourth order autoregressive model. EMG signals are described as linear combination of previous samples plus a white noise error term , using the Levinson-Durbin algorithm.

9 *Third Autoregressive (AR) coefficient:*

Third coefficient of the fourth order autoregressive model. EMG signals are described as linear combination of previous samples plus a white noise error term, using the Levinson-Durbin algorithm.

10 *Fourth Autoregressive (AR) coefficient:*

Fourth coefficient of the fourth order autoregressive model. EMG signals are described as linear combination of previous samples plus a white noise error term, using the Levinson-Durbin algorithm.

11 *Median first Autoregressive (AR) coefficient:*

Considering moving windows (length=256 samples and hop = 32 samples), it is the median of all the first AR coefficients estimated within each window.

12 *Median Absolute Deviation (MAD) first Autoregressive (AR) coefficient:*

Considering moving windows (length=256 samples and hop = 32 samples), it is the median absolute deviation of all the first AR coefficients estimated within each window.

13 *Kurtosis first Autoregressive (AR) coefficient:*

Considering moving windows (length=256 samples and hop = 32 samples), it is the kurtosis of all the first AR coefficients estimated within each window.

14 *Skewness first Autoregressive (AR) coefficient:*

Considering moving windows (length=256 samples and hop = 32 samples), it is the skewness of all the first AR coefficients estimated within each window.

15 *Median second Autoregressive (AR) coefficient:*

Considering moving windows (length=256 samples and hop = 32 samples), it is the median of all the second AR coefficients estimated within each window.

16 *Median Absolute Deviation (MAD) second Autoregressive (AR) coefficient:*

Considering moving windows (length=256 samples and hop = 32 samples), it is the median absolute deviation of all the second AR coefficients estimated within each window.

17 *Kurtosis second Autoregressive (AR) coefficient:*

Considering moving windows (length=256 samples and hop = 32 samples), it is the kurtosis of all the second AR coefficients estimated within each window.

18 *Skewness second Autoregressive (AR) coefficient:*

Considering moving windows (length=256 samples and hop = 32 samples), it is the skewness of all the second AR coefficients estimated within each window.

19 *Median third Autoregressive (AR) coefficient:*

Considering moving windows (length=256 samples and hop = 32 samples), it is the median of all the third AR coefficients estimated within each window.

20 *Median Absolute Deviation (MAD) third Autoregressive (AR) coefficient:*

Considering moving windows (length=256 samples and hop = 32 samples), it is the median absolute deviation of all the third AR coefficients estimated within each window.

21 *Kurtosis third Autoregressive (AR) coefficient:*

Considering moving windows (length=256 samples and hop = 32 samples), it is the kurtosis of all the third AR coefficients estimated within each window.

22 *Skewness third Autoregressive (AR) coefficient:*

Considering moving windows (length=256 samples and hop = 32 samples), it is the skewness of all the third AR coefficients estimated within each window.

23 *Median fourth Autoregressive (AR) coefficient:*

Considering moving windows (length=256 samples and hop = 32 samples), it is the median of all the fourth AR coefficients estimated within each window.

24 *Median Absolute Deviation (MAD) fourth Autoregressive (AR) coefficient:*

Considering moving windows (length=256 samples and hop = 32 samples), it is the median absolute deviation of all the fourth AR coefficients estimated within each window.

25 *Kurtosis fourth Autoregressive (AR) coefficient:*

Considering moving windows (length=256 samples and hop = 32 samples), it is the kurtosis of all the fourth AR coefficients estimated within each window.

26 *Skewness fourth Autoregressive (AR) coefficient:*

Considering moving windows (length=256 samples and hop = 32 samples), it is the skewness of all the fourth AR coefficients estimated within each window.

27 *Waveform length of the signals (WL):*

Cumulative length of the waveform over the time segment (Phinyomark et al., 2009).

28 *Median Waveform length of the signals:*

Considering moving windows (length=256 samples and hop = 32 samples), it is the median value of the waveform length of the signals (WL) estimated within each window.

29 *Median Absolute Deviation (MAD) Waveform length of the signals:*

Considering moving windows (length=256 samples and hop = 32 samples), it is the median absolute deviation value of the waveform length of the signals (WL) estimated within each window.

30 *Kurtosis Waveform length of the signals:*

Considering moving windows (length=256 samples and hop = 32 samples), it is the kurtosis of the waveform length of the signals (WL) estimated within each window.

31 *Skewness Waveform length of the signals:*

Considering moving windows (length=256 samples and hop = 32 samples), it is the skewness of the waveform length of the signals (WL) estimated within each window.

32 *Slope sign change (SSC):*

Number of changes between positive and negative slope among three consecutive segments (Phinyomark et al., 2009).

33 *Median Slope sign change:*

Considering moving windows (length=256 samples and hop = 32 samples), it is the median value of the slope sign change (SSC) estimated within each window.

34 *Median Absolute Deviaton (MAD) Slope sign change:*

Considering moving windows (length=256 samples and hop = 32 samples), it is the median absolute deviation of the slope sign change (SSC) estimated within each window.

35 *Kurtosis Slope sign change:*

Considering moving windows (length=256 samples and hop = 32 samples), it is the kurtosis of the slope sign change (SSC) estimated within each window.

36 *Skewness Slope sign change:*

Considering moving windows (length=256 samples and hop = 32 samples), it is the skewness of the slope sign change (SSC) estimated within each window.

37 *Zero Crossing (ZC):*

Number of times that the amplitude value of EMG signal crosses the zero y-axis (Phinyomark et al., 2009).

38 *Median Zero Crossing:*

Considering moving windows (length=256 samples and hop = 32 samples), it is the median value of the zero crossing (ZC) estimated within each window.

39 *Median Absolute Deviation (MAD) Zero Crossing:*

Considering moving windows (length=256 samples and hop = 32 samples), it is the median absolute deviation of the zero crossing (ZC) estimated within each window.

40 *Kurtosis Zero Crossing:*

Considering moving windows (length=256 samples and hop = 32 samples), it is kurtosis of the zero crossing (ZC) estimated within each window.

41 *Skewness Zero Crossing:*

Considering moving windows (length=256 samples and hop = 32 samples), it is the skewness of the zero crossing (ZC) estimated within each window.

42 *IAV (Integral of absolute value):*

Integral of the rectified EMG signal.

43 *Median IAV:*

Considering moving windows (length=256 samples and hop = 32 samples), it is the median of all the IAV feature values estimated within each window.

44 *Median Absolute Deviation (MAD) IAV:*

Considering moving windows (length=256 samples and hop = 32 samples), it is the median absolute deviation of all the IAV feature values estimated within each window.

45 *Kurtosis IAV:*

Considering moving windows (length=256 samples and hop = 32 samples), it is the kurtosis of all the IAV feature values estimated within each window.

46 *Skewness IAV:*

Considering moving windows (length=256 samples and hop = 32 samples), it is the skewness of all the IAV feature values estimated within each window.

47 *MAV (Mean Absolute Value):*

Mean value of the rectified EMG signal (Phinyomark et al., 2009).

48 *Median MAV:*

Considering moving windows (length=256 samples and hop = 32 samples), it is the median of all the MAV feature values estimated within each window.

49 *Median Absolute Deviation (MAD) MAV:*

Considering moving windows (length=256 samples and hop = 32 samples), it is the median absolute deviation of all the MAV feature values estimated within each window.

50 *Kurtosis MAV:*

Considering moving windows (length=256 samples and hop = 32 samples), it is the kurtosis of all the MAV feature values estimated within each window.

51 *Skewness MAV:*

Considering moving windows (length=256 samples and hop = 32 samples), it is the skewness of all the MAV feature values estimated within each window.

52 *Root Mean Square (RMS):*

Root mean square value of the EMG signal.

53 *Median RMS:*

Considering moving windows (length=256 samples and hop = 32 samples), it is the median of all the RMS feature values estimated within each window.

54 *Median Absolute Deviation (MAD) RMS:*

Considering moving windows (length=256 samples and hop = 32 samples), it is the median absolute deviation of all the RMS feature values estimated within each window.

55 *Kurtosis RMS:*

Considering moving windows (length=256 samples and hop = 32 samples), it is the kurtosis of all the RMS feature values estimated within each window.

56 *Skewness RMS:*

Considering moving windows (length=256 samples and hop = 32 samples), it is the skewness of all the RMS feature values estimated within each window.

57 *Integrated EMG (IEMG):*

Integral of the EMG signal.

58 *Median Integrated EMG (IEMG):*

Considering moving windows (length=256 samples and hop = 32 samples), it is the median value of the median integrated EMG (IEMG) values estimated within each window.

59 *Median Absolute Deviation (MAD) Integrated EMG (IEMG):*

Considering moving windows (length=256 samples and hop = 32 samples), it is the median absolute deviation of the median integrated EMG (IEMG) values estimated within each window.

60 *Kurtosis Integrated EMG (IEMG):*

Considering moving windows (length=256 samples and hop = 32 samples), it is the kurtosis of the median integrated EMG (IEMG) values estimated within each window.

61 *Skewness Integrated EMG (IEMG):*

Considering moving windows (length=256 samples and hop = 32 samples), it is the skewness of the median integrated EMG (IEMG) values estimated within each window.

62 *Modified MAV (Mean Absolute Value):*

It is an extension of the MAV previously described, where a weighting window function is used as reported in (Phinyomark et al., 2009).

63 *Median Modified MAV:*

Considering moving windows (length=256 samples and hop = 32 samples), it is the median value of all the modified MAV feature values estimated within each window.

64 *Median Absolute Deviation (MAD) Modified MAV:*

Considering moving windows (length=256 samples and hop = 32 samples), it is the median absolute deviation of all the modified MAV feature values estimated within each window.

65 *Kurtosis Modified MAV:*

Considering moving windows (length=256 samples and hop = 32 samples), it is the kurtosis of all the modified MAV feature values estimated within each window.

66 *Skewness Modified MAV:*

Considering moving windows (length=256 samples and hop = 32 samples), it is the skewness of all the modified MAV feature values estimated within each window.

67 *Modified MAV 2:*

It is an extension of the MAV previously described, where a weighting window function is used as reported in (Phinyomark et al., 2009).

68 *Median Modified MAV 2:*

Considering moving windows (length=256 samples and hop = 32 samples), it is the median value of all the modified MAV 2 feature values estimated within each window.

69 *Median Absolute Deviation (MAD) Modified MAV 2:*

Considering moving windows (length=256 samples and hop = 32 samples), it is the median absolute deviation of all the modified MAV 2 feature values estimated within each window.

70 *Kurtosis Modified MAV 2:*

Considering moving windows (length=256 samples and hop = 32 samples), it is the kurtosis of all the modified MAV 2 feature values estimated within each window.

71 *Skewness Modified MAV 2:*

Considering moving windows (length=256 samples and hop = 32 samples), it is the skewness of all the modified MAV 2 feature values estimated within each window.

72 *Simple Square-Integral (SSI):*

It is an estimation of the signal energy, calculated as sum of the squared signal.

73 *Median SSI:*

Considering moving windows (length=256 samples and hop = 32 samples), it is the median value of all the simple squared-integral (SSI) feature values estimated within each window.

74 *Median Absolute Deviation (MAD) SSI:*

Considering moving windows (length=256 samples and hop = 32 samples), it is the median absolute deviation of all the simple squared-integral (SSI) feature values estimated within each window.

75 *Kurtosis SSI:*

Considering moving windows (length=256 samples and hop = 32 samples), it is the kurtosis of all the simple squared-integral (SSI) feature values estimated within each window.

76 *Skewness SSI:*

Considering moving windows (length=256 samples and hop = 32 samples), it is the skewness of all the simple squared-integral (SSI) feature values estimated within each window.

77 *Willison Amplitude (WAMP):*

Number of times for each change in the EMG signal amplitude that exceeds a predefined threshold (50 mV) (Tkach et al., 2010).

78 *Median WAMP:*

Considering moving windows (length=256 samples and hop = 32 samples), it is the median value of the Willison Amplitude (WAMP) estimated within each window.

79 *Median Absolute Deviation (MAD) WAMP:*

Considering moving windows (length=256 samples and hop = 32 samples), it is the median absolute deviation of the Willison Amplitude (WAMP) estimated within each window.

80 *Kurtosis WAMP:*

Considering moving windows (length=256 samples and hop = 32 samples), it is the kurtosis of the Willison Amplitude (WAMP) estimated within each window.

81 *Skewness WAMP:*

Considering moving windows (length=256 samples and hop = 32 samples), it is the skewness of the Willison Amplitude (WAMP) estimated within each window.

82 *Variance:*

Variance of the EMG signal.

References:

Phinyomark, A., Limsakul, C., & Phukpattaranont, P. (2009). A novel feature extraction for robust EMG pattern recognition. Journal of Computing 1, 71-80.

Sinderby, C., Lindstrom, L., & Grassino, A. E. (1995). Automatic assessment of electromyogram quality. Journal of Applied Physiology, 79(5), 1803-1815.

Tkach, D., Huang, H., & Kuiken, T. A. (2010). Research study of stability of time-domain features for electromyographic pattern recognition. J Neuroeng Rehabil, 7, 21.

**Supplementary File 1M: Rank accuracy values for 1 to 10 PCs**

|  | *Kinematic synergy model* | |  | *Individual digit model* | |  | *Muscle synergy model* | |
| --- | --- | --- | --- | --- | --- | --- | --- | --- |
| # of PCs | Accuracy | SD |  | Accuracy | SD |  | Accuracy | SD |
| 1 | 80.98 | 3.96 |  | 71.32 | 4.5 |  | 62.52 | 8.98 |
| 2 | 87.33 | 3.76 |  | 84.52 | 5.76 |  | 70.67 | 7.84 |
| 3 | 89.27 | 3.71 |  | 84.85 | 5.54 |  | 71.33 | 7.75 |
| 4 | 90.59 | 3.45 |  | 85.06 | 5.79 |  | 71.57 | 8.02 |
| 5 | 91.15 | 3.63 |  | 85.86 | 5.02 |  | 72.01 | 7.70 |
| 6 | 91.21 | 3.74 |  |  |  |  | 72.58 | 7.99 |
| 7 | 91.31 | 3.67 |  |  |  |  | 73.23 | 8.28 |
| 8 | 91.56 | 3.54 |  |  |  |  | 73.09 | 8.49 |
| 9 | 91.54 | 3.59 |  |  |  |  | 73.16 | 8.36 |
| 10 | 91.59 | 3.54 |  |  |  |  | 73.20 | 8.04 |

**Supplementary File 1N: Group synergies defined by constrained *k*-means**

| *Group synergy 1* | |  | *Group synergy 2* | |  | *Group synergy 3* | |
| --- | --- | --- | --- | --- | --- | --- | --- |
| *Subject* | *Synergy* |  | *Subject* | *Synergy* |  | *Subject* | *Synergy* |
| 1 | **1** |  | 1 | **2** |  | 1 | **3** |
| 2 | **1** |  | 2 | **2** |  | 2 | **3** |
| 3 | **1** |  | 3 | **2** |  | 3 | **3** |
| 4 | **1** |  | 4 | **2** |  | 4 | **3** |
| 5 | **2** |  | 5 | **3** |  | 5 | **1** |
| 6 | **1** |  | 6 | **2** |  | 6 | **3** |
| 7 | **2** |  | 7 | **1** |  | 7 | **3** |
| 8 | **1** |  | 8 | **2** |  | 8 | **3** |
| 9 | **1** |  | 9 | **2** |  | 9 | **3** |
